# A Functional Radiological and Soft Tissue Classification to Predict Outcomes in Orbital Fracture Surgery in a Multidisciplinary “Real-World” Setting

**DOI:** 10.3389/fsurg.2021.693607

**Published:** 2021-07-27

**Authors:** Elizabeth Yang, Shu-Yi Claire Chan, Yara Al-Omari, Louise Ward, Timothy E. Yap, Aneka Jhass, Ravi Pancholi, Ahmad Aziz, Christopher Richard Bentley, Michael Perry, Vickie Lee

**Affiliations:** ^1^Ophthalmology Department, London North West University Healthcare Trust, London, United Kingdom; ^2^Imperial College London, London, United Kingdom; ^3^Imperial College Healthcare National Health Service (NHS) Trust, London, United Kingdom

**Keywords:** orbital fractures, orbital fracture ORIF, blow-out fracture of the orbit, CT analysis, orbital reconstruction, trauma

## Abstract

**Purpose:** The decision for open reduction and internal fixation (ORIF) of orbital fractures is usually based on clinical severity and soft tissue and bony findings. This study aimed to identify prognostic factors for a successful surgical outcome.

**Materials and Methods:** We included all orbital fractures treated by ORIF referred to the Ophthalmology clinic for assessment over a 12-year period. A successful outcome was defined as (i) a single operation, (ii) improved diplopia and globe position at 6 months, (iii) no surgical complications, and (iv) patient satisfaction. Data was collected on presenting symptoms, orthoptic measurements, time interval from injury to surgery, fracture geometry and involvement of internal, and external bony landmarks. Univariate and multivariate regression was used to identify predictive factors for success.

**Results:** There were 143 cases with median age 35.4 years and 81.8% (117/143) male. 51% (73/143) were complex fractures involving multiple orbital walls. 63.6% (91/143) achieved significant improvement in both enophthalmos and diplopia at 6 months. 15.3% (22/143) had significant preoperative soft tissue or neurogenic injury. 11.8% (17/143) required orbital plate repositioning or removal. 1.4% (2/143) developed orbital haematoma and 4.2% (6/143) had cicatricial entropion. Pre-operative nerve or muscle damage (OR 0.05, *p* = 0.01) and infraorbital fissure fracture (OR 0.38, *p* = 0.04) were associated with poor outcomes, whereas an intact posterior ledge was associated with successful outcomes (OR 3.03, *p* = 0.02).

**Conclusion:** Careful ocular motility evaluation to ascertain neurogenic injury and muscle compartment syndrome, and radiological analysis of the integrity of the posterior ledge and the inferior orbital fissure can facilitate management and expectations of ORIF surgery.

## Introduction

The most common cause of orbital fractures is blunt impact to the face and eye and can vary widely in extent and severity. The fractures can involve single or multiple orbital walls, the orbital rim, and extend beyond the orbit to include other fractures of the facial skeleton. Clinical presentations can also vary widely. Diplopia can occur due to mechanical restriction or neurogenic injury. Other common clinical findings include enophthalmos, vertical ocular dystopia, altered facial sensation from disturbance in the distribution of the infra orbital nerve. The decision whether to proceed with orbital repair is influenced by the severity of symptoms, the size and location of fractures, co-existing ocular injury, and patient choice following informed discussion of risks and benefits.

A “successful” result from surgical intervention in the peer reviewed literature has been variably defined and may encompass one or more of the following: (i) total resolution of symptoms, (ii) acceptable resolution or significant improvement in symptoms, (iii) patient satisfaction, (iv) absence of complications, and (v) post-operative radiographic features showing anatomical restitution from internal fixation. Many series in the peer reviewed literature are also selective for the type of fractures (many involving either the medial wall/floor). Whilst the ideal outcome is total restoration of pre-injury function and appearance, in a “real world” setting this is not always attainable in every patient, and is dependent on the extent of the initial trauma. It is possible however to achieve satisfactory outcomes in terms of function and cosmesis. This aim of this study is to analyse our outcomes of orbital fracture repair surgery, and attempt to identify and quantify any preoperative predictive factors.

## Methods

This is a retrospective cohort study of consecutive orbital fracture surgical patients referred to the Ophthalmology clinic in the multidisciplinary Orbital Trauma pathway, under the joint care of the Oral and Maxillofacial Surgery (OMFS) and Ophthalmology services at Northwest London University Hospitals (1), over a 12 year period from 2007 to 2019. This was registered and approved by London North West University Healthcare NHS Trust's Audit department. All patients underwent full orthoptic assessment including measurements of motility with Hess Chart and Field of Binocular Single vision at every visit. All patients received a full ophthalmic examination including dilated fundoscopy at least once in their care.

Our inclusion criteria were: all patients of any age with fracture(s) involving one or more walls of the orbital cavity confirmed on CT imaging, referred to our joint orbital trauma MDT pathway, who subsequently underwent open reduction and internal fixation (ORIF) of their fracture(s). Our exclusion criteria were: patients who had no evidence of orbital fracture on CT imaging, patients who did not undergo ORIF and patients with inadequate records or follow up of <6 months.

This study adhered to the tenets of the Declaration of Helsinki. We examined the medical charts of all patients with a definitive diagnosis of orbital fracture who underwent surgical repair.

We collected the following data:

1) Patient demographics (age, gender)
2) Timing of injury and surgical repair
3) Pre- and post- operative clinical findings, including diplopia, pain on eye movements, and presence of enophthalmos
4) Orthoptic eye movement measurements using the Binocular Vision Analyser [Assaf Ocular Motility Analyser (OMA; Medical Digital, Ltd., Winslow, Buckinghamshire, UK) or Thomson Software Solutions Hatfield Herts UK]. We manually quantified the Hess chart area using the method described by Aylward et al. (2) and measured the maximal vertical and horizontal deviations field of binocular single vision (BSV).
5) Preoperative radiological findings and classification. Two independent image graders (EY and YAO analysed all CT imaging, with an inter-rater reliability of 86.6%). All discrepancies were identified and taken for arbitration by one of the senior authors (MP). Analysis involved identification of:
  i) Number of orbital walls involved in the fracture
  ii) The size of orbital floor fracture (expressed as < or >50% of the floor)
  iii) Integrity of the inferior orbital fissure boundaries,
  iv) Disruption to the posterior medial bulge
  v) Integrity of the posterior ledge (such that it would not support an implant)
  vi) Involvement/displacement of the orbital rim and other orbital walls
  vii) Vertical location of the inferior recti muscle viewed on the coronal views (above, level with, or below) in relation to the orbital floor fracture defect
6) Outcome of surgery including complications, return to theatre, and need for further surgery.

We defined a successful surgical outcome if patients

i) Underwent a single procedure,
ii) With improvement or resolution of enophthalmos and diplopia (Gorman 0: no diplopia or Gorman 1 or 2: intermittent diplopia/diplopia only in the extreme gaze as successful, Gorman 3: constant diplopia as unsuccessful) by 6 months post-operatively (3).
iii) Patient satisfaction as recorded in the notes/ telephone survey
iv) Absence of complications
v) No return to theatre within 6 months.

### Surgical Technique

The standard surgical technique involved a posterior approach via a retroseptal transconjunctival incision, with minimal eyelid dissection. A lateral canthotomy was also undertaken to improve access if required. A medial transcaruncular incision was undertaken for large medial wall fractures or if fractures extended near to the orbital roof. Where there were significant periocular lacerations in some cases surgical access was via these lacerations. Sub periosteal dissection was then carried out, to formally identify and define the inferior orbital rim, inferior orbital fissure, posterior ledge, and to obtain full 360° exposure of the defect.

The contents of the inferior orbital fissure were divided as necessary (after haemostasis) to improve access to the deeper orbit.

Whenever encountered, the infraorbital nerve was protected and the anterior and posterior ethmoid or arteries cauterised. In most cases, an standard preformed titanium orbital plate was used for the orbital reconstruction. In a few cases (4.5%) a custom designed implant (for complex defects) or PDS sheet (for small defects) was used. All metal implants were secured using one or two screws placed anteriorly, either medially or laterally along the anterior edge of the implant, but always within the orbit. Trimming of the plate both posteriorly (to avoid over extension) and anteriorly (to avoid metalwork passing over the rim and risk exposure via the incision) was undertaken in most “off the shelf” cases. Thus, the implant was entirely within the orbit. Forced duction testing (vertical and horizontal directions) and clinical assessment of globe position was performed prior to closure. Wound closure with sutures was generally not required with the exception of closure of any lateral canthotomy and suspension of the anterior midface (in cases requiring zygomatic complex repair). These exceptions were sutured using standard deep/skin interrupted suture techniques.

In cases where the zygoma was significantly displaced usually involving the frontozygomatic and intra-oral buttress, an exploration of the orbital floor and ORIF was undertaken where indicated. ORIF of the infraorbital rim was only undertaken if this was significantly displaced.

### Post-operative Follow Up

All surgical patients were followed up by the OMFS department for a period of at least 6 months. Ophthalmic follow up was usually offered only to patients at high risk of persistent ocular motility or ocular pathology. Patients were contacted by telephone in June 2020 and asked whether they were willing to participate in a follow up consultation. If they gave consent then they were asked whether they had impaired vision, numbness on the affected side and whether they noted diplopia or a change in appearance after injury. They were also asked to comment on general satisfaction.

### Statistical Methods

Data were compiled and analysed using SPSS Version 25.0 (SPSS Inc., Chicago, IL, USA). We analysed pre-operative clinical orthoptic and imaging variables that would most likely influence surgical outcome and complications.

Descriptive statistics of the study population demographics and fracture characteristics were calculated using mean ± standard deviation for normally distributed data, median ± interquartile range for non-normally distributed data, and percentages for proportional data as a proportion of the total number of eyes in the study. The association of individual clinical, anatomical, and functional characteristics with post-operative success was assessed using univariate logistic regression, with Bonferroni correction to calculate an adjusted *p*-value. Multivariate logistic regression was used to model successful surgical outcome as a function of strongly performing variables in univariate regression. Variables were assessed for multicollinearity using variance inflation factor (VIF). Data were analysed using GraphPad Prism version 9.0.0 for Macintosh (GraphPad Software, San Diego, California, USA). A *p*-value or adjusted *p* < 0.05 was considered statistically significant throughout.

## Results

One hundred forty-three (30.1%) of 474 orbital fracture patients underwent ORIF. The patients who did not undergo surgery had no significant restrictive diplopia, soft tissue entrapment or enophthalmos, with minimal/no fracture displacement, were not suitable for surgery (due to globe rupture or significant ocular injury, or medically unfit for general anaesthesia), or who declined surgery. One hundred twenty-five patients (87.4%) had full pre-operative orthoptic measurements.

### Patient Demographics and Presenting Features

The median age was 35.4 (range 10.6–85.8) years and 81.8% (117/143) were male. 52.4% (75/143) involved the left orbit and 81/120 underwent repair within 30 (range 0–946) days of injury (Table 1).

**Table 1:** Demographics of study patients and presenting characteristics.

	**Age (years)**
Mean (*SD*)	38.7 (15.7)
Median	35.4
Min: Max	10.6: 85.8
**Gender**, ***n*****(%)**	
Male	117 (81.8%)
Female	26 (18.2%)
**Orbital fracture characteristics**	
Right orbit	68 (47.6%)
Left orbit	75 (52.4%)
**Time from injury to surgery (days)**	
Early (<10)	12 (8.4%)
Medium (10–30)	69 (48.3%)
Late (>30)	39 (27.3%)
Min; Max	0; 946
**Presenting symptoms and signs**	
Enophthalmos	96 (67.1%)
Diplopia	108 (75.5%)
Infraorbital anaesthesia	85 (59.4%)
Pain on eye movements	37 (25.9%)
Oculocardiac reflex	5 (3.5%)

Most late repairs were due to late presentations or had concurrent injuries (e.g., globe rupture or major systemic injuries) that required treatment before their fracture surgery. The most common presenting symptoms were diplopia (75.5%, 108/143), enophthalmos (67.1%, 96/143), and infraorbital anaesthesia (59.4%, 85/143). We did not find that the presence of these presenting symptoms was associated with a successful outcome post-ORIF.

51% (73/143) were complex fractures of more than one wall. The orbital floor was the most commonly fractured wall in our cohort (Table 2).

**Table 2:** Number of orbital walls involved in fracture.

	**Total: 143 orbital fractures**
Single wall	70 (49.0%)
Floor	46 (32.2%)
Medial wall	20 (14.0%)
Lateral wall	3 (2.1%)
Roof	1 (0.7%)
2-wall	50 (35.0%)
Floor and medial	30 (21.0%)
Floor and lateral	20 (14.0%)
3-wall	20 (14.0%)
Floor, medial and lateral	19 (13.3%)
Floor, lateral and roof	1 (0.7%)
4-wall	3 (2.1%)

### Outcomes of Surgery

63.6% (91/143) achieved a completely successful outcome, with a single surgical repair and no significant post-operative diplopia or enophthalmos at 6-month clinical review with good patient satisfaction.

22.4% (32/143) had persistent or worsened diplopia at 6 months. 68.8% (22/32) of this poor outcome group had known preoperative neurogenic injury or muscle compartment syndromes including 4 third nerve palsies (4), 3 traumatic optic neuropathies and one fourth nerve palsy. Four patients had inferior rectus orbital compartment syndrome characterised by preoperative complete upgaze restriction and severe pain. The diagnosis of neurogenic palsies was verified by a consultant ophthalmologist and orthoptists after detailed ocular motility assessment including Hess chart and field of binocular single vision. Two (1.4%) patients had persistent intractable diplopia and underwent extraocular muscle surgery (Inverse Knapp procedure and Scott's procedure). 11.8% (17/143) had to return to theatre for: orbital plate position repositioning (10 patients), orbital abscess drainage (1 patient), haematoma evacuation (2 patients), plate removal (2 patients), and 3 for further facial fracture reconstruction at 6 months after their initial surgery. Two (1.4%) patients who developed post-operative orbital haematoma: one haematoma presented and was evacuated on the same day and recovered normal vision. The other had delayed haemorrhage that occurred after discharge and did not recover vision despite undergoing urgent surgery on re-admission. Six (4.2%) developed lower eyelid cicatricial entropion and underwent entropion correction. These patients with post-operative entropion all had complex multiple-wall fracture repairs and all had orbital rim ORIF.

### Long Term Follow Up >12 Months

26% (37/143) patients were contacted and consented for a telephone follow up in June 2020 at a mean follow up of 27.7 (10–72) months with 78% operated on more than 12 months earlier. 95% (35/37) of patients reported satisfactory eyesight post-operatively. 38% (14/37) of patients reported intermittent diplopia, and 3% (1/37) experiencing constant diplopia. The most common problem reported was persistent numbness of the cheek or lip on the affected side 57% (21/37). All patients were asked if the lasting symptoms interfered with their day-to-day lives, and all reported that they were able to manage their symptoms and continue daily activities.

### Univariate Analysis on Predictive Factors of Surgical Success

We found that the severity of enophthalmos and the time interval between injury and surgery did not have any statistically significant impact on surgical success on univariate analysis (Table 3). We found that significant ocular motility restriction as measured using Hess (OR 0.997, *p* < 0.001) and BSV (OR 1.03 horizontal value, and OR 1.05 vertical value, *p* < 0.001), as well as preoperative neurogenic or extraocular muscle damage (OR 0.03, *p* < 0.001) were associated with an adverse outcome (Table 3).

**Table 3:** Influence of pre-operative factors on a successful surgical outcome.

	**Category**	**Unadjusted odds ratio for favourable outcome**	**95% confidence interval (lower limit)**	**95% confidence interval (upper limit)**	***p*-value**	**Bonferrroni correction**
**Clinical variables**						
Time to surgery	10–30 days	1.43	0.42	4.83	0.56	
Time to surgery	>30 days	0.86	0.25	3.01	0.81	
Enophthalmos	Absent or present	0.75	0.35	1.62	0.47	
Infraorbital anaesthesia	Absent or present	0.62	0.29	1.30	0.20	
Pain on eye movements	Absent or present	0.77	0.36	1.65	0.50	
Diplopia	Absent or present	0.57	0.24	1.33	0.19	
Oculocardiac reflex	Absent or present	2.20	0.24	20.25	0.49	
**Orthoptic variables**						
Hess chart	Per greater assigned points*	0.997	0.996	0.999	0.00087*	Yes
BSV: horizontal (degrees)	Per 1-degree increase	1.03	1.01	1.04	0.0011*	Yes
BSV: vertical (degrees)	Per 1-degree increase	1.05	1.03	1.08	0.0000011*	Yes
Presumed traumatic nerve or muscle damage	Present or absent	0.03	0.00	0.27	0.0015*	Yes
**Pre-operative fracture anatomy**						
Number of walls fracture	Single wall to 4-wall	0.49	0.31	0.77	0.002*	Yes
Floor	Fracture involving floor present or absent	1.53	0.39	5.98	0.54	
>50% of orbital floor fracture	>50% or <50%	0.57	0.28	1.15	0.12	
Medial	Fracture involving medial wall present or absent	0.32	0.16	0.66	0.0020*	Yes
Lateral	Fracture involving lateral wall present or absent	0.58	0.28	1.20	0.14	
Roof	Fracture involving orbital roof present or absent	0.20	0.04	1.06	0.06	
Inferior orbital fissure	Fracture involving inferior orbital fissure present or absent	0.31	0.15	0.64	0.0015*	Yes
Orbital rim	Fracture involving orbital rim present or absent	0.48	0.24	0.98	0.044*	
Zygoma	Fracture involving zygoma present or absent	0.61	0.30	1.23	0.17	
Posterior ledge*	Fracture involving posterior ledge present or absent	3.84	1.79	8.23	0.00054*	Yes
Recti muscle lying through fracture plane	Vs. lying below fracture plane	2.08	0.41	10.53	0.37	
Recti muscle lying above fracture plane	Vs. lying below fracture plane	4.02	0.90	17.95	0.07	
Posterior medial bulge	Fracture involving posterior medial bulge present or absent	0.36	0.17	0.74	0.001*	
Satisfactory implant position	Satisfactory or unsatisfactory	30.57	6.62	141.20	0.000011*	Yes

**Figure 1:**
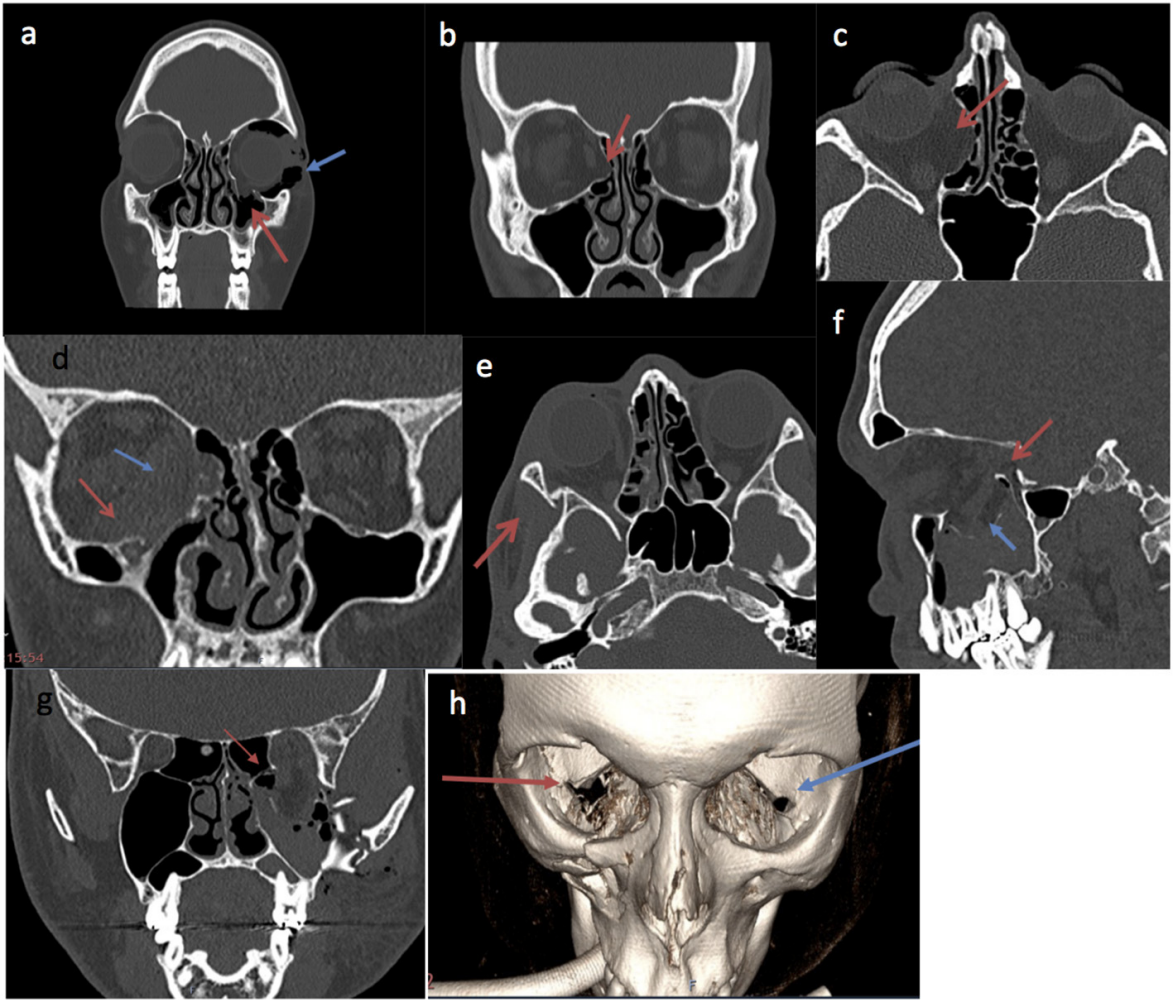
Pre-operative CT imaging examples of fracture parameters used in analysis. **(a)** Coronal slice of facial bones CT showing an isolated left orbital floor fracture (red arrow) associated with orbital emphysema (blue arrow). **(b)** Coronal slice of a facial bone CT demonstrating isolated right medial orbital wall fracture (red arrow). **(c)** Axial slice of a right isolated medial wall fracture on CT orbit (red arrow). **(d)** Coronal slice of facial bones CT showing right medial wall and right floor fracture. The red arrow points to a right orbital floor fracture. The blue arrow demonstrates right medial wall fracture. **(e)** Right lateral wall fracture (red arrow) on CT orbits. **(f)** Sagittal slice of a facial bones CT image demonstrating orbital floor fracture of >50% (blue arrow). The red arrow shows insufficient intact posterior ledge enough to support a plate, making surgical repair more challenging. **(g)** Coronal slice of facial bones CT showing left orbital floor and medial wall fracture. The red arrow demonstrates the involvement of the posterior medial bulge. **(h)** 3D image reconstruction of facial bones CT, the red arrow shows the involved inferior orbital fissure of the right orbital fracture. The blue arrow shows the intact left inferior orbital fissure.

**Figure 2:**
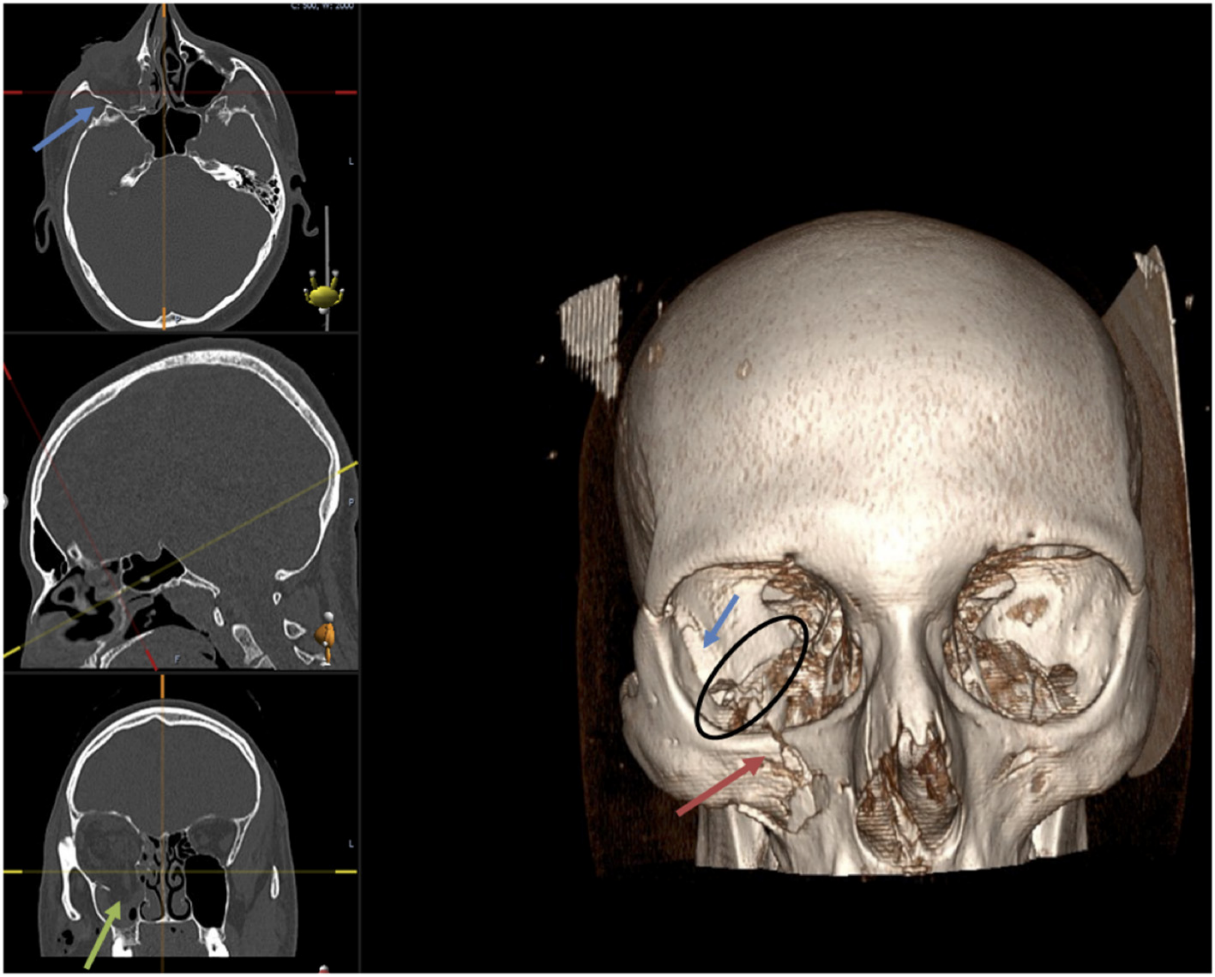
Facial bones CT demonstrating right orbital floor fracture (green arrow), lateral wall fracture (blue arrow) with the involvement of the infra-orbital rim (red arrow). The black circle shows the inferior orbital fissure (IOF) involved in the fracture on the right side compared to the left orbit.

Table detailing clinical, orthoptic, and fracture variables included into univariate logistic regression model as predictors of defined surgical success. Statistical significance determined at p < 0.05, marked^*^. Bonferroni correction was used reduce statistical errors of multiple comparisons. The Aylward system is used in the numeric grading of Hess chart deviations. Horizontal BSV is the maximal horizontal dimension in degrees, and vertical BSV is the maximal vertical dimension in degrees. Fracture anatomy parameters are demonstrated in Figures 1, 2. Fracture anatomy descriptions are based on pre-operative CT imaging.

On univariate analysis, factors associated with a poor outcome included a greater number of orbital walls involved in the fracture (OR 0.49, *p* = 0.00), fractures of the medial wall (OR 0.32, *p* = 0.00), inferior orbital fissure (OR 0.31, *p* = 0.00), orbital rim (OR 0.48, *p* = 0.44), posterior medial bulge (OR 0.36, *p* = 0.01), and posterior ledge (OR 3.84 for intact posterior ledge in the odds for success, *p* = 0.00). The size of the orbital floor fracture (> or <50%) did not significantly correlate with success (OR 0.57, *p* = 0.12; Table 3). Figures 1, 2 outline all these anatomical parameters analysed.

**Figure 3:**
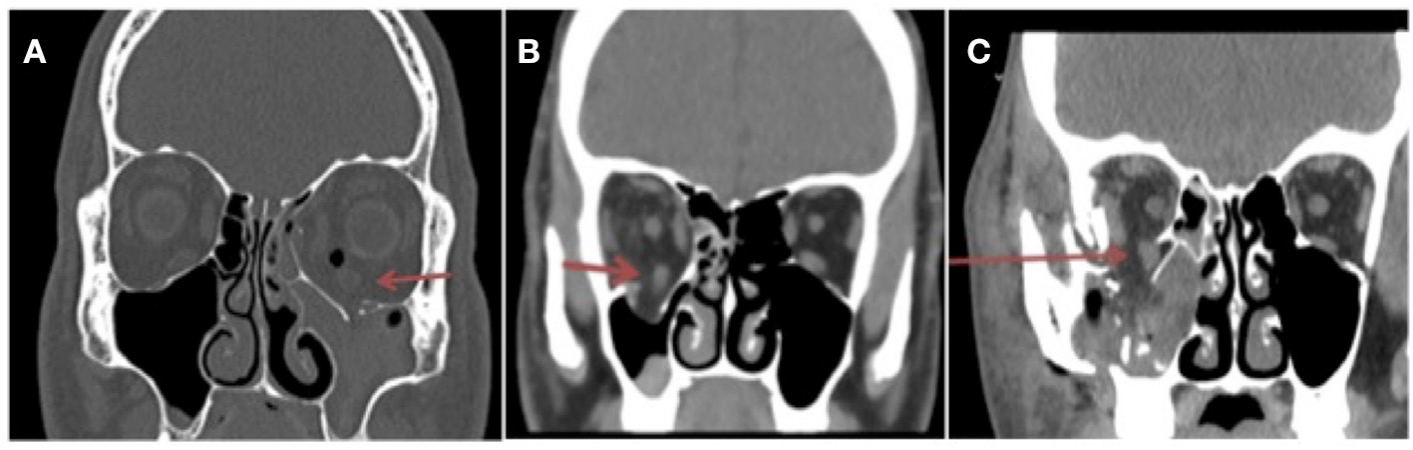
Coronal slice of facial bones CT demonstrating left orbital floor and medial wall fractures. Inferior rectus muscle is positioned **(A)** above the fracture plane (red arrow). The left maxillary sinus is obliterated and filled with blood. **(B)** The inferior rectus muscle is lying through the imaginary fracture plane. **(C)** The inferior rectus muscle falls below the fracture plane (red arrow) into the maxillary sinus.

We also noted on the coronal slices on each orbital CT image if the recti muscles adjacent to the fracture wall appeared to fall above, through, or below the fracture plane, as shown in examples in Figure 3. However, on univariate analysis, the position of the recti muscles in relation to the fracture plane did not appear to significantly correlate with success (OR 2.08, *p* = 0.37).

### Adjusted Effect of Surgical Timing, Preoperative Assessments, and Fracture Anatomy on Successful Outcome

On multivariate linear regression model (Table 4). there were three factors significantly predicted surgical success: pre-operative nerve or muscle damage predicted an unsuccessful outcome (OR 0.05, *p* = 0.01), a fractured infraorbital fissure also predicted an unsuccessful outcome (OR 0.38, *p* = 0.04), and an intact posterior ledge predicted a successful outcome (OR 3.03, *p* = 0.02).

**Table 4:** Adjusted effect variables on successful outcome at 6 months.

**Predictor**	**Category**	**Adjusted odds ratio for favourable outcome**	**95% confidence interval (lower limit)**	**95% confidence interval (upper limit)**	***p*-value**
Pre-operative Hess chart quantification	Per greater assigned points ±	0.67	0.36	1.22	0.19
BSV: horizontal (degrees)	Per 1-degree increase ±	1.01	0.51	1.99	0.97
BSV: vertical (degrees)	Per 1-degree increase ±	1.46	0.76	2.88	0.26
Pre-operative nerve or muscle damage	Absent or present	0.05	0.002	0.33	0.01*
Number of walls involved in fracture	1–4 walls	0.98	0.40	2.45	0.96
Medial wall	Fractured or not	0.42	0.10	1.64	0.22
Infraorbital fissure	Fractured or not	0.38	0.14	0.99	0.04*
Posterior ledge	Intact or not	3.03	1.22	7.70	0.02*

Results from multivariate linear regression analysis. Statistical significance determined at p < 0.05, marked*.

## Discussion

This study is a “real world” study of orbital fractures of varying complexities based in a large regional orbito-facial trauma pathway. This makes detailed analysis and robust conclusions difficult to achieve compared to most other peer reviewed studies that mainly examined single wall orbital “blow out” fractures. Our study skew toward complex fractures (51%), as only the fractures that were deemed at higher risk of orbital and ocular involvement were referred on to the multi-disciplinary pathway.

Not all orbital fractures need to be repaired surgically, and the decision for surgery is guided by the severity of double vision, enophthalmos, and the disruption of the facial skeleton. The presence of a significant ocular injury would normally be a relative contraindication for early ORIF, particularly if it were a penetrating globe injury.

Our definition of “success” is strict, as it involves both resolution of diplopia and enophthalmos, with no operative complications or return to theatre, and patient satisfaction at 6 months follow up. Most other studies tend to consider either diplopia or enophthalmos individually as single isolated endpoints. We believe our definition of success is a practical benchmark of success in the real world, as this outcome is what patients realistically would like to know.

We achieved an overall total success rate of 91 out of 143 patients (63.6%). Our results are comparable to a recent paper (5) with a similar case mix. Factors that influence the outcome of surgery are closely linked to the severity of the orbital injury. Orbital fractures represent the bony aspect of the injury, and it is important to also take into account soft tissue and neurogenic and vascular injury when considering patient outcomes.

We propose a functional and repeatable fracture classification, in line with our methodology, as a means of quantifying the level of bony injury:

i) Number of orbital walls involved in the fracture
ii) The size of orbital floor fracture (expressed as < or >50% of the floor)
iii) Integrity of the inferior orbital fissure boundaries,
iv) Disruption to the posterior medial bulge
v) Integrity of the posterior ledge (such that it would not support an implant)
vi) Involvement/displacement of the orbital rim and other orbital walls
vii) Vertical location of the inferior recti muscle viewed on the coronal views (above level with or below) in relation to the orbital floor fracture defect.

Identifying these anatomical landmarks on CT imaging is important, notably the inferior orbital fissure and the posterior ledge, which as we have shown, can predict successful surgical outcome. These structures are useful to support and align the titanium orbital implants we use. Due to the complex geometry of preformed or custom titanium implants (as opposed to flat sheets of titanium, silastic, medpor, bone, or PDS), even minor errors in orientation are less forgiving in terms of precise reconstruction of orbital geometry. In our opinion this accounts for our rate of orbital plate adjustment/removal (2 plate removals, 10 plate adjustments).

We recommend that ocular motility measurements using the Hess chart and binocular field of single vision are essential to diagnose preoperative neurogenic injury as well as significant muscle damage so that patients' expectations can be managed. We have shown here that larger pre-operative Hess chart and BSV deviations are associated with more post-operative diplopia and enophthalmos. Post-operatively, we recommend that all patients perform duction exercises as a form of ocular movement physiotherapy. Another challenging post-operative complication is the 4.2% incidence of post-operative cicatricial entropion. Our findings were similar to that of North et al. (5), usually associated with complex fractures, and we have found the treatment of these entropions challenging. As a result, we have avoided using fixation plates on the orbital rim to decrease the risk of posterior eyelid lamellar scarring.

It is reassuring that the risk of visual loss from ORIF is low. Our incidence of retrobulbar haematoma was 1.4% and visual loss 0.7% from a delayed retrobulbar haemorrhage. We recommend that it important to manage patient expectations, as perfect ocular motility outcomes are not possible in every case. In our experience, intraoperative release of entrapped soft tissues can variably affect the ocular balance. However, over the ensuing weeks or months most patients adapt to a new ocular balance and the symptoms of double vision often subside. Recovery may be incomplete due to residual scarring and/or persistent nerve weakness and some of these patients may have residual double vision when they look to the extremes of gaze. However, only a small proportion of patients (1.4%) are left with intractable diplopia in the primary and reading positions requiring ocular muscle surgery. It is also important to remember that diplopia is not a binary variable. Many previous studies have addressed this as such we feel that this should be both subjectively (Gorman score) and objectively (6) quantified.

We agree with Zimmerer et al. (7) that preoperative soft tissue morbidity is an extremely important predictor of functional outcome independent of accurate anatomical and volume restoration. Several studies have examined muscle tenting, deformity or herniation was associated with post-operative diplopia. Matic et al. have also described the rounding of the inferior rectus as a predictor of late enophthalmos (8).

The prognostic value of soft tissue herniation volume after repair of orbital floor fractures was investigated in several studies. Associations of the herniation volume with enophthalmos (9, 10), as well as with persistent diplopia and globe motility restrictions (11) have also been reported. Other similar studies are summarised in Table 5 (6, 7, 12–19).

**Table 5:** Summary of orbital fracture repair studies and outcomes.

**Authors**	**Study**	**Findings**	**Orbital implants**	**Classification of fracture/Measurement of motility**
Brucoli	75 patients, surgically managed Mean follow-up 39 months Retrospective single centre case series Isolated orbital blow-out fractures	Only significant prognostic factor was time interval between trauma and surgery (P0.05) diplopia was present in 42.5% post-op at 39 months 27.5% had persistent enophthalmos 55% had persistent infraorbital sensation disturbance	Titanium mesh (first choice) Tutopatch (some) Bone graft (complex)	No specific fracture classification system used No measurements of ocular motility detailed
Hartwig	53 surgically managed patients Mean follow-up 23 months Retrospective single centre case series Pure orbital floor fractures	Median surgery 3 days post-injury Sensitivity of the infraorbital nerve was impaired in 49.1% Diplopia was present preoperatively in 43.4% and post-operatively in 22.6% + 7% who developed diplopia after surgery Enophthalmos present in 22.6% of patients post-op Limitations in ocular motility reduced from 37.7 to 7.5% after surgery. 9.4% developed new motility restrictions post-op	PDS/Ethisorb	No specific fracture classification system used Ocular motility: Hess screen test
Higashino	107 orbits 18 surgical, 89 conservative Retrospective single centre case series 34 complex—medial and floor 73 simple—involving only medial or floor	Many recommendations regarding indications for surgery dependent on the presence of diplopia, fracture size and linearity, and degree of inferior rectus prolapse Follow-up time not stated and results are mostly for non-operative patients. No other complications mentioned	Not specified	Fracture classification: Fracture width and degree of protrusion of the inferior rectus muscle into the maxillary sinus No measurements of ocular motility detailed
Schouman	48 patients 20 surgical, 28 conservative Retrospective single centre case series Pure orbital floor fractures	Severity of inferior rectus muscle displacement is the most important independent predictive radiologic factor whether surgery needed Post-op complications not assessed	Not specified	Fracture classification: Ratio of fractured orbital floor maximal height of periorbital tissue herniation and 4-grade muscular subscore describing the position of the inferior rectus muscle relative to the level of the orbital floor No measurements of ocular motility detailed
Zimmerer	Prospective multicentre study 144 patients, all surgically managed *Post-hoc* analysis Both simple and complex fractures	64.6% favourable outcome Diplopia in in 24.3% of patients 4.2% orbital asymmetry without diplopia. 3.5% both diplopia and orbital symmetry. Authors recommend the radiological study not sufficient to predict outcome due to soft tissue factors	Titanium plates from different manufacturers	Fracture classification: AOCMF Level 3 classification Ocular motility: “Follow my finger” test—patient asked to follow examiner's finger in 8 directions
Choi	63 conservatively treated patients Retrospective single centre case series Simple fractures—multiple wall fractures excluded	Defect area 0.98 cm^2^ and herniated fat volumes of 343.40 mm^3^ predictive of late enophthalmos at 2 months 49.2% had enophthalmos at 2 months	N/A—not surgically managed	No ocular motility measurements Measurement of herniated fat volumes and defect area using CT cross-sectional imaging
Jung	181 surgically managed patients Retrospective single centre case series Both simple and complex fractures	EOM tenting and deformity and ratio of volume of herniated orbital soft tissue to fracture size were found to be statistically significant risk factors of diplopia 40.9% had diplopia at 6 months post-ORIF Post-op rates of enophthalmos and ocular motility disturbance not stated	Not specified	Fracture location, fracture type (closed or open flap),fracture size & volume of herniated orbital soft tissue, ratio of volume of herniated orbital soft tissue to fracture size, number of points of contact between EOM and bony edge, presence of EOM thickening, displacement, deformity testing and entrapment, and EOM swelling ratio Ocular motility measured but method not specified
Harris	30 surgically managed patients Retrospective single centre case series Orbital floor fractures with or without extension into medial wall	Patients with soft tissue distortion relative to bone fragment configuration tend to have poor post-operative outcomes Only ocular motility measured—no other post-op complications recorded	Nylamid implant	Measured BSV Improvement Measured relationship between preoperative soft tissue disruption on coronal CT images and post-operative ocular motility
Furuta	113 patients 64 surgical, 49 conservative Retrospective single centre case series Pure blowout fractures only	Number of points of muscle contact with orbital fracture site on CT is an important indicator of preoperative and post-operative extraocular muscle function	Bone fragments or hydroxyapatite plates	Ocular motility: Hess Area Ratios Classification: Number of points of contact of EOMs to fracture edge and whether fracture was open or closed flap on CT
Senese	79 ORIF Retrospective single centre case series Orbital floor 52 Orbital floor and medial wall 22	Majority had surgery within 14 days 21% diplopia 29% infraorbital anaesthesia 13% enophthalmos	PDS and titanium	No clear fracture classification system used Presence/absence of diplopia No orthoptic measurements

*EOM, extra-ocular muscle; ORIF, open reduction and internal fixation; PDS, polydioxanone*.

Interestingly, we found no association between timing of surgery and success. There have been published suggested benchmarks of timely orbital fracture repair being within 2 weeks of presentation (20). Being a tertiary referral centre, we receive patients who present late for a variety of reasons. We include a cohort of “elective” ORIFs, where 18 of these patients were initially minimally symptomatic from diplopia and developed late enophthalmos, and therefore underwent late ORIF. Equally we are skewed toward more severe injuries, especially with cases requiring secondary revision. 16.1% of our cohort had 3 or 4-wall fractures with multiple facial bony injury, who underwent concurrent ORIF alongside other facial reconstruction surgery, typically after stabilisation of more life-threatening injuries. These patients tended to present much later than 2 weeks after their initial injury, and often require further revision procedures.

Our study is limited by its retrospective nature and inclusion of patients cared by multiple surgeons with limited follow up. It is also dependent on the quality of medical record keeping—the severity of subjective diplopia using the Gorman score was not noted in many records. A small minority of 14.3% did not have preoperative Hess chart and BSV field measured and often these patients were referred from OMFS to Ophthalmology after their surgery. Our patients also range in complexity and variety of orbital and facial fractures and are managed in a tertiary referral centre.

In conclusion, we recommend that all patients with orbital fracture repair should undergo full ophthalmic examination and ocular motility measurements including Hess charts and BSV assessments to identify prognostic soft tissue injury. Surgeons should also consider a functional CT orbit imaging classification that we have described, to identify anatomical prognostic factors for the likelihood of surgical success.

## Data Availability Statement

The raw data supporting the conclusions of this article will be made available by the authors, without undue reservation.

## Ethics Statement

Ethical review and approval was not required for the study on human participants in accordance with the local legislation and institutional requirements. Written informed consent for participation was not required for this study in accordance with the national legislation and the institutional requirements.

## Author Contributions

EY conceptualised the project, analysed the data, and wrote the manuscript together with S-YC. S-YC, YA-O, LW, and RP collected the data for the project. TY assisted with statistical analysis. VL and MP conceptualised the project and provided supervision together with AA and CB. All authors reviewed the final manuscript prior to submission.

## Conflict of Interest

The authors declare that the research was conducted in the absence of any commercial or financial relationships that could be construed as a potential conflict of interest.

## Publisher's Note

All claims expressed in this article are solely those of the authors and do not necessarily represent those of their affiliated organizations, or those of the publisher, the editors and the reviewers. Any product that may be evaluated in this article, or claim that may be made by its manufacturer, is not guaranteed or endorsed by the publisher.
